# Structure and Function of the Human Ryanodine Receptors and Their Association with Myopathies—Present State, Challenges, and Perspectives

**DOI:** 10.3390/molecules25184040

**Published:** 2020-09-04

**Authors:** Vladena Bauerová-Hlinková, Dominika Hajdúchová, Jacob A. Bauer

**Affiliations:** Institute of Molecular Biology, Slovak Academy of Sciences, Dúbravská Cesta 21, 845 51 Bratislava, Slovakia; hajduchovadominika@gmail.com (D.H.); jacob.bauer@savba.sk (J.A.B.)

**Keywords:** ryanodine receptor, mutation, crystal structure, cryo-electron microscopy, molecular dynamics, normal mode analysis, cardiac arrhythmias, myopathy

## Abstract

Cardiac arrhythmias are serious, life-threatening diseases associated with the dysregulation of Ca${}^{2+}$ influx into the cytoplasm of cardiomyocytes. This dysregulation often arises from dysfunction of ryanodine receptor 2 (RyR2), the principal Ca${}^{2+}$ release channel. Dysfunction of RyR1, the skeletal muscle isoform, also results in less severe, but also potentially life-threatening syndromes. The *RYR2* and *RYR1* genes have been found to harbor three main mutation “hot spots”, where mutations change the channel structure, its interdomain interface properties, its interactions with its binding partners, or its dynamics. In all cases, the result is a defective release of Ca${}^{2+}$ ions from the sarcoplasmic reticulum into the myocyte cytoplasm. Here, we provide an overview of the most frequent diseases resulting from mutations to RyR1 and RyR2, briefly review some of the recent experimental structural work on these two molecules, detail some of the computational work describing their dynamics, and summarize the known changes to the structure and function of these receptors with particular emphasis on their N-terminal, central, and channel domains.

## 1. Introduction

Ryanodine receptors (RyRs) are the largest ion channels presently known. They are very large (2.2 MDa) Ca${}^{2+}$ channels that play a key role in excitation-contraction (EC) coupling [1,2,3,4,5]. RyRs are present in all animals [6,7]. Three RyR isoforms have been identified in mammals: RyR1, RyR2 and RyR3. RyR1, the most studied of them [7,8,9,10,11,12], is primarily expressed in skeletal muscle [8]. RyR2 is predominantly expressed in cardiac muscle [13,14], but is also present in smooth muscle and in neuronal tissue [15,16]. RyR3 is expressed at low levels in a variety of tissues, including the nervous system and the muscle of the diaphragm [15,17]. The amino-acid sequence identity between all three isoforms is 66%. The largest differences are clustered in three “divergent regions”, which include residues 1342–1403, 1872–1923, and 4254–4631 (RyR1 numbering). Mouse knock-out experiments have shown that RyR1 and RyR2 have a critical role in physiology and development [18,19]: mice deficient in RyR1 died perinatally due to respiratory failure while those lacking RyR2 died during the early stages of embryonic development. Mice missing RyR3 were viable [20,21,22] but did exhibit impaired social behavior [21,23,24]. In non-mammalian vertebrates, for example chickens or ferrets, only two RyR isoforms have been identified, typically designated RyRα and RyRβ [25]; lower organisms appear to express only one isoform [26].

Structurally, RyRs are homotetramers, with each subunit having around 5000 amino acids. They are most often found embedded in the sarcoplasmic reticulum (SR) membrane of striated muscle cells at t-tubule triad junctions [27]. At these junctions, they are situated adjacent to L-type voltage-gated calcium channels, also called 1,4-dihydroxypyridine receptors (LCCs or DHPRs). DHPRs are composed of five subunits, though only two regions are essential for excitation-contraction coupling: the II–III loop of the $\alpha_{1S}$ subunit [28,29,30,31,32,33] and the C-terminal tail of the $\beta_{1a}$ subunit [33,34,35]. EC coupling refers to the close interaction between DHPRs and RyRs in which depolarization of the SR membrane is coupled to the opening of the RyRs [1,7,36]. Two distinct mechanisms by which RyR opening is triggered and Ca${}^{2+}$ is released into the cytoplasm from the SR have been described. In cardiomyocytes, during membrane depolarization, the DHPRs open, allowing small amounts of Ca${}^{2+}$ to pass from the extracellular space into the myocyte cytoplasm. These Ca${}^{2+}$ ions then bind to high-affinity sites on the RyR, thereby activating it [37,38,39,40,41,42] and increasing the likelihood that it will open [43]. RyR opening releases the Ca${}^{2+}$ stored in the SR into the cytoplasm. These ions then go on to bind to the troponin C in myofilaments, thereby initiating muscle contraction [44,45,46]. The process by which Ca${}^{2+}$ ions bind the RyR, causing it to open and release additional calcium, is termed calcium-induced calcium release, and it and its many regulatory mechanisms have been extensively studied on the physiological, molecular and biophysical levels [2,47,48,49]. In skeletal muscles, RyRs are directly linked to DHPRs [28,50]. In this situation, two components of the DHPR interact with RyR1 in response to depolarization of the surface membrane. The sensitivity of DHPR to voltage changes at the plasma membrane results in a conformational change that is sensed by RyR1 [7].

RyRs are regulated and influenced by a large number of factors [3,51,52]. The most important of these is the cytosolic Ca${}^{2+}$ concentration itself. A plot of Ca${}^{2+}$ conductance versus Ca${}^{2+}$ concentration has a bell shape, showing that RyR is activated by the presence of Ca${}^{2+}$, but inhibited by higher concentrations of it [53]. It is generally believed that Ca${}^{2+}$ binds to high affinity Ca${}^{2+}$-binding sites in RyR, thereby raising the probability of opening. When the Ca${}^{2+}$ concentration is high enough, other, lower affinity sites become occupied, increasing the probability of channel closing [51,52,54,55]. The sarcoplasmic ATPase then reduces the cytosolic Ca${}^{2+}$ concentration by pumping Ca${}^{2+}$ ions back into the SR [56]. Other regulating or influencing factors include calmodulin (CaM) in both its *apo* and Ca${}^{2+}$-bound forms, FKBP12 and FKBP12.6, protein kinase A (PKA), calmodulin kinase II (CaMKII), DHPR, calsequestrin, triadin, and junction as well as Mg${}^{2+}$, ATP, and caffeine [3]. RyRs have also been shown to be influenced by oxidative species and phosphorylation [57,58].

Mutations that disrupt the functioning of the RyRs are often associated with myopathies. In the following sections, we will outline the most frequent diseases associated with RyR1 and RyR2 and then review the results of recent structural and computational studies with an emphasis on how individual mutations might disrupt either the structure or the regulation of one of the receptors.

## 2. Diseases Associated with RyR1 and RyR2

**Figure 1:**
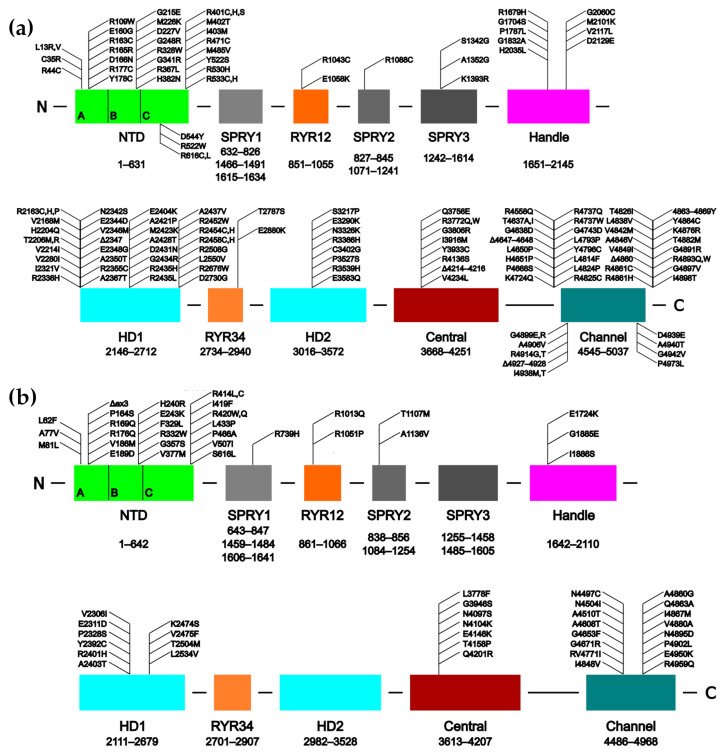
Schematics of the human RyR1 (**a**) and RyR2 (**b**) sequences showing the domain organization and the approximate locations of several disease-causing mutations. The individual subdomains of the N-Terminal Domain (NTD) are indicated. Δex3 refers to the CPVT1-associated deletion of exon 3. The SPRY domains are comprised from more than one region of the sequence, hence their multiple sequence ranges. Adapted from Bauerová-Hlinková et al. [103].

To date, over 450 mutations have been identified in the gene of *RYR1*, which are associated with life-threatening myopathies (these are listed in the Human Gene Mutation Database, http://www.hgmd.cf.ac.uk/ac/gene.php?gene=RYR1 [59]; Figure 1a). The largest number of these mutations, nearly 200, are associated with malignant hyperthermia (MH) [60,61,62,63,64,65]. The next most frequent myopathies are central core disease (CCD) [65,66,67,68,69,70,71], congenital myopathy [72,73,74,75] and multi-minicore disease (MMD) [65,71,76,77,78].

Over 250 mutations associated with cardiomyopathies have been identified in the *RYR2* gene (listed at http://www.hgmd.cf.ac.uk/ac/gene.php?gene=RYR2; Figure 1b). The vast majority of them are associated with catecholaminergic polymorphic ventricular tachycardia, type 1 (CPVT1) [79,80]. Other diseases that are caused by mutations to the RyR2 channel include arrhythmogenic right ventricular dysplasia, type 2 (ARVC/D2) [81,82,83,84], polymorphic ventricular tachycardia (PVT) [85,86], sudden infant death syndrome (SIDS) [87], sudden cardiac death (SCD) [88,89,90,91,92], and left ventricular non-compaction (LVNC) [93,94,95,96].

The molecular mechanisms by which these mutations affect the functioning of the RyR2 channel are still not clear, but it is known that their presence disrupts the correct opening and closing of the channel [97,98]. Without medical intervention, CPVT1, ARVC/D2 and SCD lead to fatal arrhythmias, which can be triggered by physical strain or emotional stress. Presently, only the symptoms of these disorders can be treated (using β-blockers, verapamil, or by implanting defibrillators) and all of these treatments are deficient in some way [99,100].

In the original studies, mutations in both isoforms tended to be found in three “mutation hot-spot” clusters. These appeared in the N-terminal region (roughly residues 1–600), the central region (roughly 2100–2500) and the C-terminal region (from around 3800 to the C-terminus) [3,7,97,101,102]. Presently, a number of RyR1 mutations have also been found which lie between these areas, but the hot-spot clusters clearly remain in RyR2 [5] (Figure 1b).

RyR3 has recently been linked to Alzheimer’s disease in a mouse model [104] and family-based association tests have shown that some RyR3 variants can be linked to hypertension, diabetes and Alzheimer’s disease in humans [105], but the details of these processes have not yet been explored. A recent study has also linked RyR3 with gender dysphoria [106].

### 2.1. Diseases of RyR1 Dysfunction

**Malignant hyperthermia** is a pharmacogenetic disorder that manifests in a sudden increase in body temperature, muscle stiffness and, in some cases, the breakdown of skeletal muscle fibers (rhabdomyolysis). It is mainly triggered by volatile anesthesia or muscle relaxants; stress can also trigger the disease in some cases [107]. Mutations that cause MH normally lead to increased release of calcium from the sarcoplasmic reticulum and intracellular calcium overload. This leads to increased binding of calcium to the myofilament, which causes dysregulated skeletal muscle contraction. This contractile state results in muscle rigidity and, eventually, skeletal myocyte breakdown. Muscle rigidity in this context results in a hypermetabolic state, causing body temperature to rise over time. Myocyte breakdown can cause elevated serum creatine kinase levels, myoglobinuria (potentially leading to renal failure), and hyperkalemia. The abnormally high Ca${}^{2+}$ level also leads to increased H${}^{+}$ and CO${}_{2}$ levels in the cell and plasma. This chain of events leads to the classic collection of symptoms seen during an MH episode: acidosis, rigidity, hypercarbia, and hyperthermia [108,109,110]. Dantrolene, a hydantoin derivative, is currently the only approved treatment for MH. Although dantrolene inhibits RyR1, with which it directly interacts [108,111,112], its mechanism of action remains unknown.

**Central core disease** is a congenital myopathy that most commonly develops during childhood and can lead to death. It is characterized by sections of muscle fiber that lack mitochondria. CCD typically manifests in infancy with hypotonia (muscle weakness) and delay in motor development and is predominantly characterized by a proximal weakness in the hip girdle; orthopedic complications are common and malignant hyperthermia susceptibility is a frequent complication. In the majority of patients, the weakness is either static or progresses only slowly, and the long-term outcome is favorable [113].

### 2.2. Diseases of RyR2 Dysfunction

**CPVT1** (Catecholaminergic Polymorphic Ventricular Tachycardia, type 1) is a ventricular tachycardia (acceleration of heart activity) which is triggered by stress. The basis of the disease is excessive Ca${}^{2+}$ leakage from the sarcoplasmic reticulum during diastole [114,115]. It is an inherited, autosomally dominant channelopathy of the heart [116]. The disease is rare, but very malignant, often leading to sudden cardiac arrest during childhood or adolescence. Five causative genes of CPVT have been reported: *RYR2* [116], *CASQ2* [117], *KCNJ2* [118], *TRDN* [119] and *CALM1* [120]. Priori et al. [116] first reported that mutations in the *RYR2* gene are associated with CPVT1. Other clinical studies have identified a large number of other mutations to the *RYR2* gene associated with CPVT1 [79,80,82,96,118,121,122,123]. Interestingly, dantrolene, which does not appear to bind to wild-type RyR2, also seems to improve the function of mutant RyR2s both in vitro [124,125], and in vivo in knock-in mice [126]. There are also several studies examining its effects on cardiomyocytes derived from induced pluripotent stem cells from human patients [127,128], and at least one clinical study showing improvement in some human CPVT patients [129]. The primary side-effects reported for dantrolene are muscle weakness and drowsiness, dizziness, general malaise, and diarrhea. It also has the potential to cause liver damage in higher doses; consequently, it is unlikely to be useful as a chronic treatment.

**ARVC/D** (Arrhythmogenic Right Ventricular Cardiomyopathy/Dysplasia) is a disease that causes sudden death in people below the age of 30. ARVC/D’s symptoms arise from arrhythmias causing a reduction in blood flow to certain organs, including the brain. It is characterized by the progressive deterioration of the right ventricle, arrhythmias and sudden cardiac death. ARVC/D2 differs in that it is associated with ventricular tachycardia triggered by excessive exertion [130,131]. Several clinical studies have linked *RYR2* mutations to ARVC/D [81,82,83,84,132,133,134]. There are presently no clinical trials exploring the effect of dantrolene on ARVC/D.

## 3. Structural Studies of the RyR1 and RyR2 Isoforms

Ryanodine receptors were first visualized by electron microscopy as periodically-repeating electron-dense areas occupying the space between the SR membrane and the transverse-tubule membranes of skeletal muscle triads [135]. Later studies succeeded in isolating and biochemically characterizing these structures and also acquired electron microscopy images of individual particles, which showed that the receptor had a square shape [136,137,138]. These were also the first studies to definitively show that the ryanodine receptor and the Ca${}^{2+}$ release channel were one and the same. These early studies established the general shape of the receptor and gave a rough idea of its size, but later cryo-EM and X-ray crystallography studies began to fully develop the details of the channel.

### 3.1. Cryo-EM Studies

**Figure 2:**
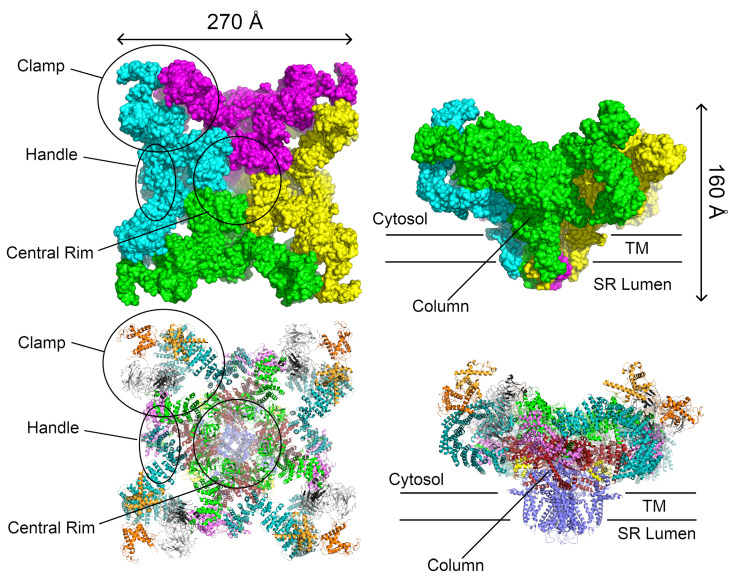
Two representations of the RyR1 tetramer. Top: The solvent-accessible surface area allows the large-scale structures originally identified by cryo-EM to be distinguished. Bottom: A ribbon diagram showing which domains correspond to the individual large-scale structures. The views to the right are rotated 90${}^{{}^{\circ}}$ about the horizontal axis. The individual domains are colored as in Figure 1 and Figure 3. The structure is the 3.8 Å RyR1 structure (PDB ID 3j8h) from Yan et al. [11]. This structure was chosen to illustrate the structural features of the ryanodine receptors because it has the highest resolution and completeness (part of the long helical domain is lacking in the available RyR2 structures).

Given the size and complexity of the oligomer, the structure of the entire channel has been determined only by electron microscopy. Initial cryo-EM studies with a resolution of ≈24 Å were performed on a closed conformation of the RyR1 isoform isolated from rabbit skeletal muscle [139]. Subsequent studies increased the resolution to ≈10 Å [140,141]. At this resolution, the tetrameric structure of these channels, with a four-fold axis of symmetry along the pore of the channel, was clearly visible. The entire RyR tetramer is composed of an extensive cytoplasmic region, comprising ≈80% of the total protein structure and measuring ≈275×275×100 Å and a smaller TM region measuring ≈120×120×60 Å; these regions are connected through a column. At this resolution, several distinct areas of the cytoplasmic region could be resolved, including the clamp, the handle, and the central rim (Figure 2); all of which play important roles in the regulation of these channels [142].

**Figure 3:**
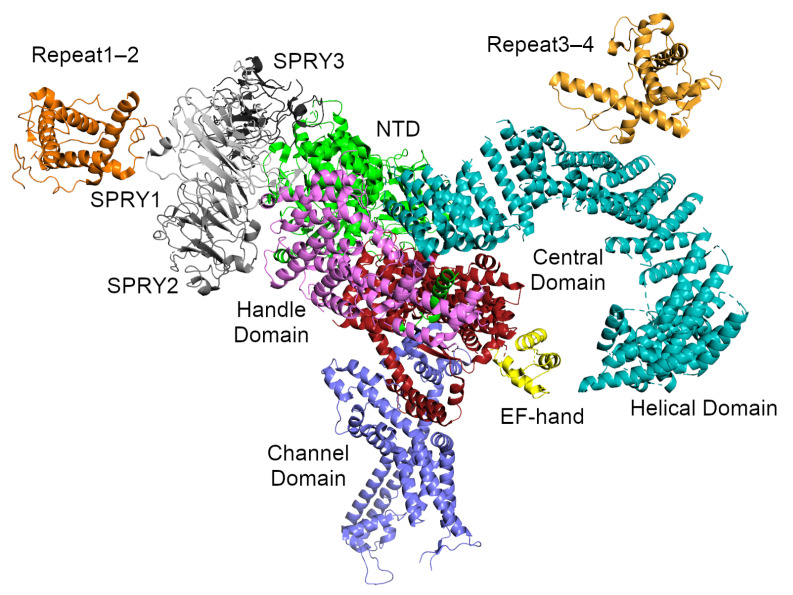
The RyR1 monomer from Yan et al. [11]. Two perpendicular α-solenoids can be distinguished. α-solenoid 1 runs top-to-bottom and includes NTD:C, part of the handle domain, and the central domain. α-solenoid 2 runs left-to-right and includes part of the handle domain and the long, curved helical domain. The SPRY domains and Repeat1–2 are attached to the periphery of solenoid 1, while the phosphorylation target domain Repeat3–4 sits atop solenoid 2. The domains are colored as follows: the NTD is green, the SPRY domains are gray (SPRY1 is light gray, SPRY2 is middle gray, SPRY3 is dark gray), Repeat1–2 is darker orange, the handle domain is magenta, the two halves of the helical domain are cyan, Repeat3–4 is lighter orange, the central domain is red, and the transmembrane channel domain is purple. The central domain of RyR1 includes a calcium-binding EF-hand motif which is colored yellow.

Several high-resolution cryo-EM structures (up to 3.8 Å) have been reported of rabbit RyR1 [10,11,12,143,144]. These structures were determined in both the closed and open states of RyR1 [12,143,144] as well as in complex with the RyR modulators FKBP12 [11], Ca${}^{2+}$ [144,145], ATP and caffeine [145]. The influence of different detergents on the conformation of the RyR1 channel in both the open and closed conformations has also been investigated [146]. At this resolution, it could clearly be seen that RyR1 was a homotetrameric channel and that each monomer was made up of ten domains: an N-terminal domain (NTD), three SPRY domains interrupted by an RyR repeat domain (Repeat1–2), a “handle” domain, an extended α-helical domain interrupted by a second RyR repeat domain (Repeat3–4), a central domain, the transmembrane channel, and a C-terminal domain (CTD; Figure 3). The size of the channel was also refined to 270×270×160 Å (Figure 2). The NTD can be further divided into three subdomains A, B, and C. Aside from the N-terminal domain and the SPRY domains, the majority of the structure is α-helical. In particular, the previously uncharacterized central, handle and helical domains have armadillo repeat folds and, together with the N-terminal domain, provide a “network of superhelical scaffold” for binding channel modulators and the propagation of conformational changes [11].

At intermediate scales, the receptor can be thought of as consisting of two extended α-solenoids at roughly right-angles to each other (Figure 3). The first α-solenoid is formed from subdomain C of the NTD together with the central domain and part of the handle domain. It consists of 30 α-helices packed into two parallel layers. This domain likely serves to support the RyR cytoplasmic region and extends to the surface of the handle region of the receptor, where it bends under the A and B subdomains of the NTD [12]. The second α-solenoid (α-solenoid 2) comprises part of the handle domain together with the long, curved Helical Domain. It runs roughly perpendicular to α-solenoid 1 and forms most of the clamp region and the square edges of the cytoplasmic part of the receptor (Figure 2). Since it forms such a large part of the surface and since the best known function of an α-solenoid domain is to bind other proteins [147], it seems likely that many of the RyR interaction partners associate here.

**Figure 4:**
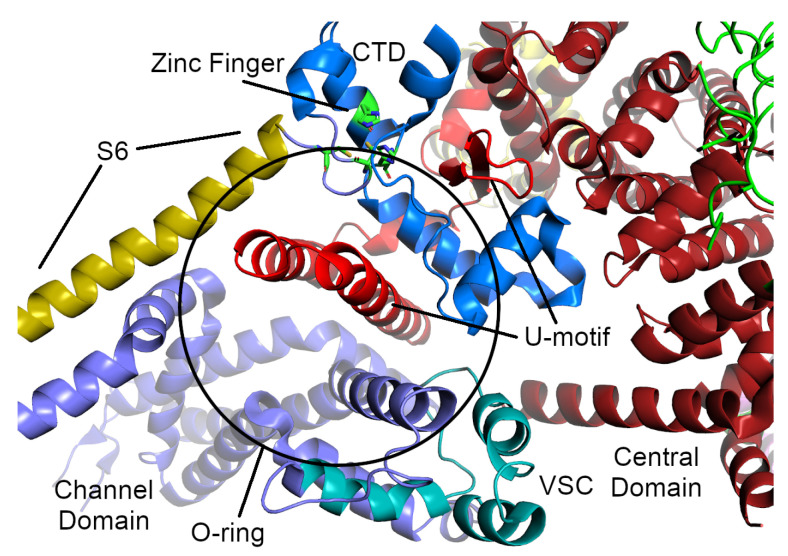
The O-ring and U-motif of the RyR1 structure from Yan et al. [11]. The U-motif from the central domain is colored a brighter red than the rest of the central domain. The parts of the O-motif are colored as follows: the cytosolic part of the S6 α-helix from the TM domain is sand-colored, the voltage-sensor-like motif is colored cyan, and the C-terminal zinc-finger domain is colored blue (the residues of the zinc-finger themselves are colored green and shown as sticks).

The transmembrane (TM) channel domain represents a chimera of voltage-gated sodium and pH-activated ion channels [12]. In particular, the transmembrane helices S1–S6 assemble into a voltage-gated ion channel superfamily fold, but one which also has three additional features. First, the S6 segment is unusually long, with half in the membrane and half in the cytoplasm. It is immediately followed by the small, α-helical CTD, which possesses a C2H2-type zinc-finger. Second, the residues between S2 and S3 fold into a cytoplasmic voltage-sensor-like domain (VSC), which bridges the CTD and S1–S4 segments. Third, the residues between S5 and the pore helix form a hairpin loop which projects into the SR lumen. The elongated S6 segments, the CTD, and the VSC together create a cytoplasmic O-ring in each monomer. The C-terminal residues of the central domain (roughly 4170–4250 in RyR1, 4134–4207 in RyR2) form a U-motif which hooks into the O-ring (Figure 4). The concave face of the α-solenoids, which lies above the U-motif, wraps around the O-ring. The VSC, CTD, and central domains together comprise the column seen in the lower resolution cryo-EM studies [4,142] (Figure 2).

Comparison of the open and closed structures shows a dilation of the S6 tetrahelical bundle at the cytoplasmic gate of the channel domain that leads to channel opening. During pore opening, the cytoplasmic “O-ring” motif of the channel domain and the U-motif of the Central domain exhibit coupled motion, while the central domain undergoes domain-wise displacement [143]. Binding sites for the channel activators Ca${}^{2+}$, ATP and caffeine were identified at the interdomain interfaces of the C-terminal domain [144,145]. Ca${}^{2+}$ binding to the EF-hand in the central domain initiates a cascade of conformational transmissions through the U-motif, downwards to the VSC, the CTD and S6, inducing S6 to change conformation, thus opening the channel. Upon Ca${}^{2+}$ binding, the EF-hand motif retains its basic fold, but undergoes pronounced conformational changes [144].

A set of high-resolution (3.6–6 Å) cryo-EM structures of porcine RyR2 have also recently been determined [148,149,150], which currently represent the highest-resolution EM structures of this isoform (an 11.8 Å structure of rabbit RyR2 in complex with FKBP12.6 has also recently been determined [151]). In these studies RyR2 was determined in both its open and closed (or *apo*-RyR2) forms [148], as well as in complex with the RyR2 allosteric regulators Ca${}^{2+}$, PCB95, ATP, caffeine, FKBP12.6 and calmodulin [149,150,151] in different combinations in order to better understand their regulation of this protein. Comparing the open and closed states of RyR2 showed that no large structural changes occurred within the cytoplasmic domains themselves, but that the overall motion of the cytoplasmic region came from domain-wise displacement and relative shifts between the domains. The central domain was found to be the primary transducer of conformational changes controlling the opening and closing of the channel domain [11,143,148]. This domain undergoes both intradomain contraction and a domain-wise rotation, which collectively results in the outward tilting of the pore-forming S6 segments.

These cryo-EM structures found that the Ca${}^{2+}$, ATP and caffeine binding sites are all located at the interfaces between the central and channel domains [150], which are identical to those found in the RyR1 structures [145]. In particular, Ca${}^{2+}$ binds at a cleft formed by the central domain and the CTD; ATP is buried in a pocket formed by the U-motif, S6, and the CTD, and caffeine is located at the interface formed by the U-motif, S4, the CTD, and the VSC. Apo-calmodulin and Ca${}^{2+}$-bound calmodulin bind to distinct but overlapping sites in an elongated cleft formed by the handle, helical and central domains [150]. Taken together, these studies are in agreement with the observation that contraction and relaxation of the central domain is critical for RyR channel gating [11,12,143]; this was also earlier found by site-directed mutagenesis experiments on the RyR2 and RyR3 isoforms [152,153].

Regarding RyR2 gating, under Ca${}^{2+}$-free conditions, RyR2 is inactive because of the absence of stimulation by cytoplasmic Ca${}^{2+}$. An increase in Ca${}^{2+}$ concentration to around 20 μM induces contraction of the central domain, applying a pulling force that facilitates the dilation of the S6 bundle. The addition of PCB95 induces further contraction of the central domain and opens the channel. In contrast, FKBP12.6 induces relaxation of the central domain, stabilizing the closed state of RyR2. Neither Ca${}^{2+}$/ATP nor Ca${}^{2+}$/caffeine were sufficient to open the channel in the presence of FKBP12.6. Caffeine keeps the central domain in a constitutive contracted state, and ATP strengthens the pulling force by bridging the U-motif and S6 [149,150]. A study combining single-channel measurements with modeling showed that the triphosphate moiety of ATP alone was able to either activate or irreversibly inactivate RyR2, but triphosphate with adenosine prevented the inactivation and showed activation similar to that of ATP [154].

Although RyR1 and RyR2 have very similar overall quaternary structure (which is expected given that they have ≈70% sequence similarity), comparing their cryo-EM structures shows substantial structural differences between the two. This is most notable in the second half of the helical domain (HD2) structure forming the clamp domain [148,151], which participates in quaternary interactions with the dihydropyridine receptor and neighboring RyRs in RyR1 but not in RyR2. Moreover, all of the RyR2 EM structures showed higher flexibility in the peripheral domains, including the two RyR domains, the three SPRY domains, and the A and B subdomains of the NTD. The effective resolution of all these domains was lower and not sufficient for detailed analysis [148,149].

### 3.2. Crystal Structures of Individual RyR Domains

Several domains from RyR1 and RyR2 have had structures determined at atomic resolution by X-ray crystallography or NMR (Table 1); together these cover ≈15% of the total sequence of this channel [5]. The structures were determined of both native and mutant forms, with the mutants being some of those associated with the myopathies described earlier. Presently, crystal structures have been determined for most of the N-terminal domain (NTD) of both RyR1 and RyR2 [155,156,157] and its individual subdomains [158,159,160,161], the RyR1 SPRY1 and RyR1 and RyR2 SPRY2 domains [162,163], the first RYR domain (Repeat1–2) from RyR1 [162], and the second RYR domain from all three RyR1, RyR2, and RyR3 isoforms (Repeat3–4) [162,164]. Short peptides of the central domain in complex with calmodulin [165] and the S100A1 protein [166] have also been determined.

#### 3.2.1. N-Terminal Domain

**Figure 5:**
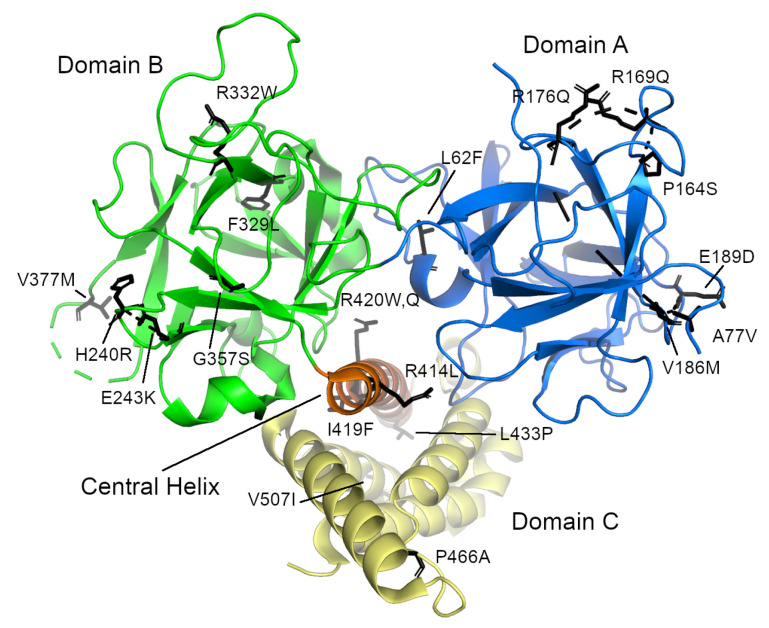
The N-terminal domain of human RyR2 from Borko et al. [157] (PDB ID 4jkq). The individual subdomains are colored blue (domain A), green (domain B), and yellow (domain C). The “central helix” is colored orange. The locations of individual disease-causing mutations are shown as sticks. Note that they are grouped in three distinct places: on the surfaces of domains A and B and on the central helix. The long, disordered loop containing residues 98–107 is not visible in this structure.

Several research groups have focused on determining and characterizing the structures of the N-terminal domain (NTD) of RyR1 [155,156] and RyR2 [157,160]. The NTD comprises three separate subdomains, commonly called A (residues 1–223 in human RyR2), B (224–408), and C (409–643). Domains A and B are β-trefoil domains and are classified as an Inositol 1,4,5-triphosphate/ryanodine receptor domain and a MIR (protein mannosyltransferase, IP3R and RyR) domain, respectively, while domain C is an all α-helical domain classified as an RIH (RyR and IP3R Homology) domain [103] and appears to be an armadillo-repeat domain. The first α-helix of domain C, called the central helix (residues 410–439), lies near the middle of the interface where these three domains come together and was proposed to play an important role in NTD stability (Figure 5) [157]. Only the first half of domain C is visible in the NTD crystal structures. SAXS and molecular dynamics simulations suggest that the second half unfolds to a greater or lesser extent in the absence of the rest of the protein [169]. It is clearly visible in the high-resolution cryo-EM structures, however, where it is sandwiched between the rest of the NTD and the handle domain (with some support also given by the central domain). Domain A of the RyR2 isoform also contains a dynamic α-helix (residues 95–104, α2) [161], which was invisible in most of the RyR2 NTD crystal structures [157,159,160] as well as in the high-resolution EM structures [148]. In the EM structure of the whole RyR2 receptor, this helix is located at the surface and its formation may depend on the binding of various interaction partners. The corresponding loop in isoforms 1 and 3 is shorter, which is consistent with the absence of this α-helix these isoforms [161]. Based on cryo-EM studies, the NTD is part of the central rim [10,11,12,148].

The NTD covers one of the so called “hot spot” areas, where mutations associated with MH [60,61,62,63,64,65] and CCD [65,66,67,68,69,70,71] in RyR1 (http://www.hgmd.cf.ac.uk/ac/gene.php?gene=RYR1) and CPVT [79,82,118,121,122] ARVC/D2 [81,132,133,134], SUO [170,171], SCD [85,118] and E-IVF [88] in RyR2 are located (http://www.hgmd.cf.ac.uk/ac/gene.php?gene=RYR2; see also http://triad.fsm.it/cardmoc/). So far, over 70 mutations associated with these diseases have been identified in RyR1 and 40 in RyR2. Consequently, both the NTD and its subdomains of both isoforms have been thoroughly examined structurally in order to better understand how exactly a particular mutation affects the tertiary structure or the opening and closing of the ryanodine receptor channel [155,156,157,158,159,160,161].

The NTD mutations in both isoforms occur in three main areas: the central helix, the interfaces between the NTD subdomains or between the other RyR domains, and the so-called HS-loop, which is located in subdomain A between β-strands 8 and 9 [155,157,158,159,160,161]. To date, there are crystal structures of the L14R, C36R, R45C, D61N, V219I, G249R, R402G, and I404M mutants of RyR1 [156] and the A77V, V186M, R420Q, P164S, R169Q, and R176Q mutants of RyR2 [159,160,161]. The RyR2 mutations I419F and R420W have also been biochemically and biophysically characterized [157]. Typically, these structures showed that the mutations caused only local structural changes, or modest rearrangements of the positions of the three N-terminal domains with respect to one another. It therefore seems that these mutations exert their effect not by distorting the structure of the protein itself, but by weakening the interfaces between domains or between the receptor and its binding partners (e.g., FKBP), thereby causing improper domain shifts during the opening and closing of the RyR channel. A number of other mutants occur within the interior of the subdomains or which are located at the interdomain interface (e.g., L62F, F329L, T415R, I419F, and L433P in RyR2) could not be prepared in a stable, soluble form [156,157], suggesting that these residues likely disrupt the structure of the NTD. In general, three types of mutants have been proposed: those that cause misfolding, those that may destabilize the interactions between the N-terminal A, B, and C domains, and those which destabilize the interfaces with the other RyR domains.

The RyR2 NTD contains a long, flexible loop in subdomain A which contains two flexible α-helices. One of these helices can be seen in the wild-type RyR2 NTD:A-only structure (PDB ID 3im5) [159], but cannot be seen in structures of the whole NTD [157,160]. This area is affected by a CPVT1- and LVNC-associated Δexon3 mutation, which deletes residues 57–91 from RyR2, and thereby removes β-strand 4 and one of the flexible α-helices from domain A. Remarkably, instead of being lethal, the remaining α-helix unwinds and becomes a β-strand, taking the place of the missing β4, and thereby largely rescuing the β-trefoil of subdomain A [161].

#### 3.2.2. SPRY1 and SPRY2 Domains

Both the α-solenoids described earlier have peripheral domains which lie on their surfaces. For α-solenoid 1, this includes three SPRY domains (SPRY1–3) as well as an RYR repeat domain (Repeat1–2); the sequence of α-solenoid 2 is interrupted by the second RYR repeat domain (Repeat3–4), which lies on the surface of the solenoid. The structures of the SPRY1 (from RyR2) and SPRY2 (from both RyR1 and RyR2) domains have been determined by X-ray crystallography with resolutions of 1.2–1.5 Å (Table 1) [162,163].

The sequences of three SPRY domains (from dual specificity kinase SplA and RyRs, where they were first found) interpenetrate one another to a limited extent: the core of the domains consists of a continual, uninterrupted stretch of amino acids, however there are extra residues at the domain peripheries that appear much later in the sequence. For example, in the 3.8 Å RyR1 structure [11], SPRY1 consists of residues 632–826 (SPRY core), 1466–1491 (an extension of the β-sheet closest to the receptor core), and 1615–1634 (an extension to the β-sheet closest to the surface); SPRY2 consists of residues 827–845 (an extension to the β-sheet closest to the surface) and 1071–1241 (SPRY core); SPRY3, uniquely, consists of two larger, structurally conjoined regions, residues 1242–1465 and 1492–1614, separated by the first SPRY1 extension. This pattern is also repeated in the RyR2 structure [148]. The extensions to SPRY1 and SPRY2 take the form of additional anti-parallel pairs of β-strands which extend one of the two SPRY domain β-sheets. Because of this domain arrangement, the individual SPRY1 and SPRY2 domains that have been solved do not cover the entire domain, but only the largest continuous segment forming the core [162,163].

**Figure 6:**
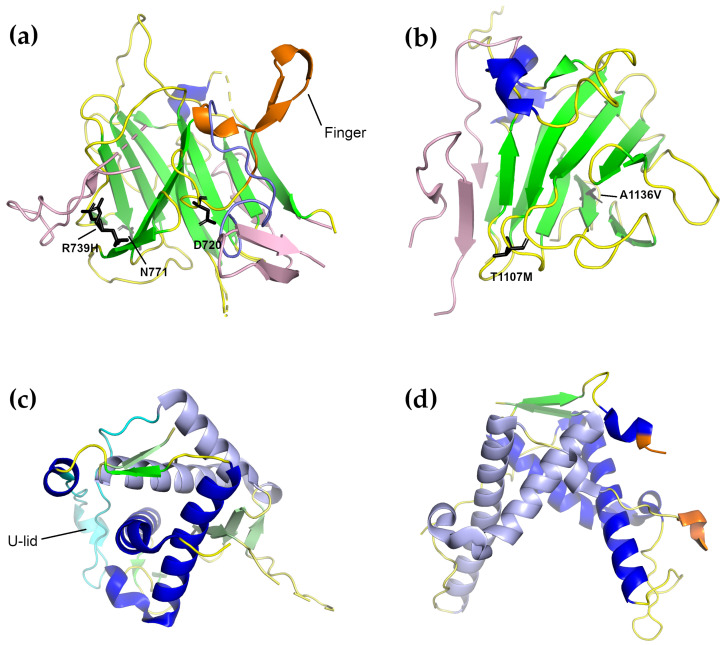
Four representative structures of the individual RyR domain crystal structures described in the text. Domains from the RyR2 isoform are shown except for Repeat1–2, where only the RyR1 structure is known. (**a**) The RyR2 SPRY1 domain (PDB ID 5c33 [162]). The β-sheet extensions present in the full-length structure but missing in the SPRY1 crystal structure are shown in pink. The “finger” motif is colored orange, while the conformation of the loop comprising the finger domain in the complete structure is colored purple. Residues of which mutations are associated with diseases in RyR2 are shown as sticks. (**b**) The RyR2 SPRY2 domain (PDB ID 4p9i [163]). Coloring is as in (**a**). In (**a**,**b**), the extensions were placed by superimposing the given structure on the closed form of the full-length RyR2 cryo-EM structure (5go9 [148]). (**c**) The RyR1 Repeat1–2 domain (PDB ID 5c30 [162]). The U-lid of this domain is colored cyan. To make it easy to distinguish the two repeats, Repeat 2 is colored a lighter shade than Repeat 1. (**d**) The RyR2 Repeat3–4 domain (PDB ID 4etv [164]). The coloring is as in (**c**). The beginning and end of the phosphorylation loop, most of which is not visible, are colored orange.

Structurally, the SPRY1 and SPRY2 domains have very similar folds, which are one of the most common β-sandwich folds found in eukaryotes (Figure 6a,b). They are often found as the central domains of ligases, the binding domains for regulatory proteins, and they facilitate the assembly of macromolecular complexes [172]. The core of both structures consists of two anti-parallel β-sheets covered by a “lid” that forms a cap over the domain core. In the complete RyR structure, both “lids” face the solvent. The RyR2 SPRY1 structure is characterized by the presence of a “finger” formed by an anti-parallel β-loop-β motif with a $3_{10}$ helix before the first β-strand and a second $3_{10}$ helix between the two strands [162]. In the complete RyR2 structure, however, this motif has collapsed into a loop which is buried under the second SPRY1 extension (residues 1606–1641), which, in turn, contacts the junction between the NTD domain C α-solenoid and the handle domain. In this context, the high conservation of the residues in this motif is likely due to its position in an interior part of the protein: disruptions here are likely to ruin the conformation of the entire three-SPRY domain unit. The SPRY2 domain structure of both RyR1 and RyR2 contained an inserted loop which interrupts β-strand 8 [163], which was not observed in the SPRY1 domain. In addition N-terminal and C-terminal extensions were also observed. In the complete structures of both RyR1 and RyR2, the inserted loop lies on the surface of the protein while the C-terminal extensions form part of the first β-strand of SPRY3. When docked into lower-resolution cryo-EM maps, SPRY2 was placed between the clamp region and the central rim (which is formed by the NTD) [163]. This position was verified in the full structures, where SPRY2 can clearly be seen to fill a gap between the handle domain of one subunit and the helical domains of the next [10,11,12,148]; its position was also verified by cross-linking experiments [12]. The SPRY2 domain is therefore likely required to couple the conformational changes in both areas, and disease-causing mutations on its surface may interfere with this process [163].

Two mutations in human RyR1 SPRY1 have previously been linked to myopathies: D708N has been linked to multi-minicore disease and atypical periodic paralysis [173], while N759D is associated with central core disease [174]. The possible effect of both mutations on the structure of the SPRY1 domain of RyR2 was analyzed in [162]. The equivalent of D708 in RyR2 is D720. In the crystal structure, D720 forms a salt bridge with the equally conserved R694. Since D720 is part of the “finger” motif, and since the “finger” motif was thought to mediate the SPRY1–SPRY2 interaction, it was thought that a mutation to asparagine would weaken the interaction and therefore disrupt the SPRY1–SPRY2 interaction. In the full structure, however, the finger motif becomes a buried loop and this interaction does not occur. D720 is, however, in a position to hydrogen-bond to the main-chain atoms of the first SPRY1 extension (1466–1491) where it arises from SPRY3, so it is possible that the D720N mutation still disrupts an interaction between two SPRY domains, but SPRY1–SPRY3 rather than SPRY1–SPRY2. This analysis also holds for the structure of the RyR1 isoform. The second mutation, N759D was clearly shown to disrupt the binding of the FKBP [162], and likely would have a similar effect in both isoforms.

In the RyR2 SPRY1 domain, only one mutation has so far been linked to CPVT1, R739H [79]. In the crystal structure of SPRY1, the side-chain of R739 is found in a different conformation than in the full-length protein. In the full-length RyR2 structure, R739 lies below the first SPRY1 extension (1459–1484) and is in a position to potentially interact with residues from SPRY3. Its replacement with histidine would still allow it to interact with the extension, but it would then be too short to reach the residues of SPRY3.

In the RyR1 SPRY2 domain, a total of five mutations have been identified: three of them are associated with CCD (R1075W, G1165D, and R1179W [75,175]) and two with malignant hyperthermia (R1127H and R1140C [62,64]). The structural impact of all of them on the RyR1 SPRY2 domain was described in Lau et al. [163]. In this case, the analysis is largely valid for both the partial-domain crystal structure and the structure in the full-length protein, with one notable exception: In the full-length structure, R1075 lies at the junction between SPRY2 and SPRY3, and an R1075W mutation in this area might be expected to disrupt this interface. Moreover, there is already a neighboring tryptophan (W1237 rabbit RyR numbering) in this area together with two proline residues (P1111 and P1609); the introduction of an extra tryptophan in this site might be enough to change the electrostatic character of the surface. G1165 lies in a tight β-turn-β motif which interacts with the “lid” and a G1165D mutation in this site would be expected to disrupt these interactions. The remaining residues are all surface exposed and likely disrupt the binding of regulatory proteins.

In the RyR2 isoform SPRY2 domain, two mutations have been identified which are associated with CPVT: T1107M and A1136V (Figure 6b) [79]. The effects of these mutations are not clear from either the crystal structure or the full-length protein. T1107 is largely surface exposed and A1136 is buried. In the former, a T1107M mutation might be expected to disrupt the binding of an effector if regulatory molecules do bind in this area. In the latter, an A → V mutation might be expected to disrupt the packing by decreasing the surface complementarity. Recently, a potentially new RyR2 mutation, P1124L, identified in a patient from a genotype-negative HCM cohort, was structurally and functionally characterized [176]. In this study, the P1124L mutation was found to trigger functional and structural alterations in isolated RyR2 channels and whole hearts. This mutation induced significant conformational changes in the SPRY2 domain of RyR2. Recombinant RyR2-P1124L channels displayed a cytosolic loss-of-function phenotype, which contrasted with a higher sensitivity to luminal Ca${}^{2+}$, indicating a luminal gain of function.

#### 3.2.3. RYR1–2 (Repeat1–2) and RYR3–4 (Repeat3–4) Domains

The ryanodine receptor contains four tandem repeats that occur in two sets of two each. These are also called RYR domains, because they have so far only been found in the ryanodine receptors [103], or sometimes phosphorylation domains because at least one of them, Repeat3–4, has been shown to contain a number of phosphorylation targets. In particular, PKA was found to phosphorylate five residues in Repeat3–4: S2808 [177,178,179], T2810, S2811, S2814, and S2797 [164]. Four residues were found that were phosphorylated by CaMKII: S2808, S2811, T2876 [164], and S2814 [164,180]. The corresponding serine residues did not appear to be phosphorylated in the RyR3 Repeat3–4 domain [164]. Each domain comprises around 200 amino-acid residues and forms a U-shaped motif [162,164,167]. In the RyR sequence, Repeat1–2 is located as an insertion in SPRY2, immediately following the SPRY2 N-terminal extension, while Repeat3–4 is an insertion in the long Helical Domain (α-solenoid 2).

Structurally, the first two RYR repeats (Repeat1–2) are composed of two asymmetrical halves (Figure 6c). Each tandem repeat of the RYR domains consists of two α-helices followed by a single short C-terminal β-strand. These strands together form a double-stranded anti-parallel β-sheet. The second tandem repeat contains an additional three-stranded β-sheet which follows the first α-helix of repeat 2 and fills the space between repeat 1 and repeat 2; this extra β-sheet disrupts the symmetry of these repeats [162]. The two repeats are separated from each other by a 30-residue loop, which forms a U-shaped lid and interacts extensively with the first α-helix of repeat 1. (It is worth mentioning here that this crystal structure is to be preferred over the structure of the corresponding domain in the 3.8 Å full-length RyR1 structure [11]. That structure was modeled de novo based on the Repeat3–4 structure and is lacking many of the structural features present in this crystal structure.) Docking this domain into the cryo-EM maps of RyR1 placed them in the corners of the cytoplasmic domain, just over the SPRY2 domain, a positioning which was confirmed by the high resolution structures [11,12,162].

The RYR3–4 domain (Repeat3–4), also referred to as the phosphorylation domain, is one of the few domains that has high resolution structures from all three mammalian isoforms [164,167]. The X-ray crystal structures of this domain show that it has a high, nearly two-fold symmetry and a horseshoe shape; it is similar in all three RyR isoforms (Figure 6d) [164]. Unlike Repeat1–2, the tandem repeats of this domain are separated by a flexible loop, which contains several serine residues that are phosphorylation targets. In particular, S2808, T2810, S2811, and S2814 are all within the phosphorylation loop; S2797 lies in a small $3_{10}$ helix immediately preceding the phosphorylation loop and is expected to be solvent-exposed in the full-length receptor. T2876 is not located in or near the phosphorylation loop, but is found in a short $3_{10}$ helix on the opposite side of the domain; in the structure of the full-length protein, this is in contact with the first half of the helical domain (HD1). In the RyR3 isoform, part of the phosphorylation loop is replaced by an α-helix. Repeat3–4 lacks the three-stranded β-sheet and the structured U-lid of Repeat1–2, and the loops connecting the α-helices also have different conformations. Docking of the Repeat3–4 domain into the cryo-EM maps of RyR1 and RyR2 revealed that this domain is part of the clamp region of the receptor [12,148,164,167,181]. The structure of the Repeat3–4 domain from the diamondback moth ryanodine receptor has also been determined. It was found to possess an additional N-terminal α-helix and to have some domain rearrangement relative to the mammalian isoforms [182].

Recently, a crystal structure of the mouse RyR2 Repeat3–4 domain bound to the catalytic domain of PKA (PKAc) in complex with the non-hydrolyzable ATP analog adenylyl-imidodiphosphate (AMP-PNP) was determined [168]. In this structure, PKAc was in a non-catalytic open conformation and the phosphorylation loop of Repeat3–4 was found to encircle the large lobe of PKAc. Binding to PKAc caused conformational changes to this region: in addition to ordering the loop, it caused the $3_{10}$ helix before the loop and the α-helix following it to unwind. A closed PKAc form was also obtained, but only in complex with a 12-residue peptide from the phosphorylation loop and not the whole repeat domain. To examine the effect of CaMKII phosphorylation at residue S2814 (S2813 in the mouse form), a complex of PKAc and RyR2 Repeat3–4 harboring a S2814D phosphorylation-mimicking mutation was also determined. It was found that a new α-helix was created at the C-terminal end of the phosphorylation loop, which resulted in enhanced interactions with PKAc.

Five mutations associated with malignant hyperthermia are found in the RyR1 Repeat1–2 domain. Four of them are located at the domain surface, and are therefore unlikely to cause domain misfolding, but one of them R1043C (R1044C in rabbit RyR1), affects a residue that is involved in multiple hydrogen-bonds, including ones to main-chain atoms from the U-lid; breaking these may therefore affect the U-lid conformation and stability [162]. Two CPVT-associated mutations are known in the RyR2 Repeat1–2 domain: R1013Q [79] and R1051P [79,122]. Since the high-resolution structure of this domain is not yet known, the structural effect of these mutations can only be inferred based on the cryo-EM structures of RyR2 [148,149,150]. R1013 in both the closed and open RyR2 conformations is located at the surface of the domain and does not appear to take part in any stabilizing interactions. R1051, however, is located in the middle of the second α-helix of repeat 2 and its replacement with proline would very likely disrupt this helix, and possibly the structure of at least part of the domain.

Eleven mutation sites associated with malignant hyperthermia, central core disease, multi-minicore disease, King–Denborough syndrome and atypic periodic paralysis have been found in the RyR1 Repeat3–4 domain (summarized in [162]). Seven of them are located within or near the phosphorylation loop, three are located on the opposite side of the molecule, though not especially close to the T2876 phosphorylation site, and the last is an L2867G mutation which lowers the melting temperature of the domain and seems to disrupt the hydrophobic core. No disease-associated mutations have yet been reported for the RyR2 Repeat3–4 domain.

## 4. Computational Studies

### 4.1. Early Bioinformatics and *In Silico* Studies of Ryanodine Receptors

Before detailed structural studies had been performed, a number of studies sought to partially characterize the structure of RyR with the help of bioinformatics. Bioinformatics studies on RyR2 focused on identifying individual domains based on their amino-acid sequences using the Pfam database [103,183]. These studies predicted that 14 domains would be found in all monomers of all three RyR isoforms. In general, the domain organization predicted in this approach agrees with the high-resolution electron microscopy studies performed on RyR1 [10,11,12] and RyR2 [148,149,150,151]. The results from these bioinformatics studies were used to design RyR2 N-terminal domain constructs which were overexpressed in *E. coli* [183], purified, and characterized on the biochemical, biophysical and structural levels [103,157,184].

Before the high-resolution cryo-EM structures had been completed, some effort was made to fit the existing high-resolution crystal structures into the low-resolution cryo-EM maps; nearly every structural study featured at least some effort to fit the studied domain into at least one of the available cryo-EM maps [155,156,157,162,163,164,182]. In one case, the placement of the RyR1 NTD subdomains by molecular dynamics flexible fitting into both the closed and open maps was confirmed using fluorescence resonance energy transfer studies [185].

### 4.2. Dynamics Studies

Determining the three-dimensional structure of the ryanodine receptor in both its open and closed forms does provide the answer to a number of questions about the architecture of the molecule and its likely conformational changes upon opening and closing, but it says little about the pathway by which the channel opens, what other conformational states are possible, and what the effects might be on the structure of the binding of the various regulatory molecules. Moreover, though the structures can be used to identify the location and possible significance of individual point mutations, by themselves, they still cannot be used to describe in detail their mechanistic consequences. To address these questions, computational studies are needed.

The first high-resolution cryo-EM structures of RyR1 were all in the closed form [10,11,12]. One of the goals of the first computational studies after these became available was to create models of the open form. The first of these published was a normal-mode analysis by Wenjun Zheng [186] on the 3.8-Å RyR1 structure. He found that the largest collective motions of the closed form involved large outward and downward movements of the peripheral domains; these were in good agreement with the conformational variations observed in multiple cryo-EM structures of the closed form that were later reported by des Georges et al. [145]. By using normal mode analysis to flexibly fit the closed form into a 10-Å cryo-EM map of the open form, he was also able to create a model of the open form of the receptor. A similar open-form conformation was created by Mowrey et al. [187] using an ion-pulling molecular dynamics simulation on only the transmembrane portion of the structure. Both models recreated the major features of the RyR1 and RyR2 open-channel structures reported subsequently, including the rotation of the S6 helix away from the channel axis.

Several computational studies have been carried out on the structure of the ryanodine receptor that combine in vitro or in vivo functional studies with dynamics modeling to assess the functional and structural impact of one or more of the known disease-associated mutations. The part of the receptor most frequently studied in this way is the pore domain, followed by the NTD. The earliest dynamics study examining the channel pore region predated the reporting of the high-resolution cryo-EM structures and used a homology model of the RyR1 pore region that was based on the pore of K${}^{+}$ channels. Schilling et al. [188,189] used molecular dynamics to examine the dynamics of Ca${}^{2+}$ ions when approaching the receptor and the effect of a number of mutations to the area on the outside of the pore on this distribution. They found that the distribution on the luminal side was most affected by the mutations G4898R, D4899Q, E4900Q, R4913E, and D4917A, all of which had previously been shown to reduce the ability of RyR1 to conduct Ca${}^{2+}$. They found that the Ca${}^{2+}$ ions are channeled by the protein surface to the luminal entry of the RyR, which allowed them to provide a plausible structural account for the experimental observations.

Three studies in the laboratory of Gerhard Meissner have examined the effect of mutating RyR1 channel residues G4934 and G4941, both of which are in the pore-lining helix S6. The first study [190] looked at the effect of substituting these residues with progressively larger amino acids on channel gating and ion permeation. They found that G4934A, G4941A, and G4941V continued to exhibit a caffeine-induced Ca${}^{2+}$ release response and bound ryanodine, but had significant differences in open times and number of gating events. Increasing the size of the side-chains still further (G4934V and G4941I) resulted in reduced caffeine-induced Ca${}^{2+}$ release, low levels of ryanodine binding, and no Ca${}^{2+}$ conductance in single-channel measurements; the Ca${}^{2+}$ regulation was also abolished. Mutant structures were created from the 3.8 Å cryo-EM RyR1 structure using Eris [191] to create the mutant structure and GROMACS [192] to optimize its geometry. The results showed that the larger substitutions decreased the protein stability by introducing clashes with neighboring residues. In a later study [193], they found that a G4941K mutation did not fully close at a 100 nM Ca${}^{2+}$ concentration and fully opened at 2 μM concentration (the wild-type channel had only a 13% probability of opening at this concentration). They prepared models of the mutant structure in the same way and found that this opened form is stabilized by the formation of a salt bridge between the mutant lysine and D4938. Their most recent study [194] explored the effect of a double deletion in RyR1 of residues F4923 and F4924 (both in S6), which were associated with a lethal form of fetal akinesia deformation syndrome in two brothers. Cellular, electrophysiological, and computational methods were used to characterize the consequences of this mutation. They found that the homotetrameric mutant channel was not activated by Ca${}^{2+}$ and did not conduct Ca${}^{2+}$, but that a heterotetrameric channel (created by expressing both the wild-type and mutant forms simultaneously in HEK cells) did result in Ca${}^{2+}$-dependent channel activation with intermediate Ca${}^{2+}$ conductances. Molecular dynamics simulations on a homology model of the homotetrameric mutant channel suggested that the mutation decreased the protein stability and abolished the salt-bridge interaction between R4944 and D4938, thereby increasing the electrostatic interaction energy in an area 20 Å away from the mutation site. Because the mutation was heterozygous, it was ultimately concluded that this double deletion alone was likely not responsible for all the observed symptoms.

In a similar study, homology modeling was combined with clinical assessment of a family suffering from CPVT and functional characterization using Ca${}^{2+}$ release experiments to characterize a newly-identified I4855M RyR2 loss-of-function mutation [96]. The homology model showed that the mutation was in an area termed the “inner vestibule,” a water-filled cavity where Ca${}^{2+}$ ions may remain in a hydrated fashion. The mutation could therefore either affect Ca${}^{2+}$ hydration or the positions of the pore helices, either of which would decrease Ca${}^{2+}$ permeability; the mutation also markedly inhibited caffeine activation of RyR2.

Finally, sequence alignment and homology modeling was used to locate and describe the site of a mutation, G4946E, that confers resistance to diamide insecticides to the diamondback moth *Plutella xylostella* [195]. The site is in the channel domain in helix S4, close to the S4-S5 linker domain and the voltage sensor-like domain.

Similar functional-modeling studies have also been carried out on the RyR1 NTD. The earliest was by Zheng and Liu [196], who simulated the tetrameric gate created by the NTD of RyR1 together with three disease mutations (K155E, R157Q, and R164Q), which lie at the interface between subdomain A in one monomer and subdomain B in the adjacent one. They found that there is a dynamic network of salt-bridges at this interface and that the three mutations disrupted it, increasing the interdomain flexibility and the probability of channel opening. A similar molecular dynamics study, reaching similar conclusions, was carried out by Xiong et al. [197] in support of a clinical and mouse study on a *de novo* A165D mutation.

Bauer et al. [169] carried out a molecular dynamics study on wild-type human RyR2 and its R414L, I419F, and R420W mutants, which were either unstable during purification or failed to crystallize during a previous structural study [157]. Their results suggested that RyR2 possesses a moderate level of flexibility, which allows the channel to close and not easily open without an opening signal. The mutations studied, however, disrupted this balance by making the gate either too rigid or too loose, causing closing to become difficult or less effective. In particular, molecular dynamics suggested that the R414L and I419F mutants, which are associated with SCD and SUO [85,170] hinder channel closing, whereas R420W, which is associated with ARVC/D2 [132,133,134], may enhance channel opening.

The most comprehensive NTD modeling study to date was by Yamazawa et al. [198], who combined functional analysis with molecular dynamics simulation of mutant NTD homology models. They found that the C36R, E40A, D61A, Q156K, R164C, R164L, G249R, G342R, R402C, R402H, Y523C, and Y523S mutations all introduced abnormalities in Ca${}^{2+}$ homeostasis. Their functional studies revealed that the mutations located around the central helix (R402C and R402H) increased the open probability of the channel. Molecular dynamics simulations of both the NTDs separately and in a tetrameric arrangement caused clockwise rotation of the B and C domains with respect to the A domain by altering the interdomain interactions (mainly hydrogen-bonds and salt-bridges). It was noted that a similar movement occurs in the wild-type during channel opening, thus the central domain movements may lead to a shift through the handle and central domains, which could ultimately increase the flexibility of S6, thereby increasing the open probability of the channel pore.

Finally, many of the structures described above, including the human RyR2 NTD (4jkq), mouse RyR2 NTD (3im6), mouse RyR2 SPRY1 (5c33), rabbit RyR1 Repeat1–2 (5c30), mouse RyR2 SPRY2 (4P9I), mouse Repeat3–4 (4etv) and rabbit RyR1 (3j8h) (Table 1), were used to construct a human RyR2 homology model. This was used in a retrospective observational cohort study of CPVT patients diagnosed below 19 years of age and their first-degree relatives [123]. The mutations identified in these patients were mapped onto this model to determine their likely structural importance. There was a clear association between cardiac arrest and mutations at the intersubunit interfaces of the NTD and the S4-S5 linker and the S5 and S6 helices of the pore domain.

## 5. Conclusions, Further Perspectives and Challenges

Ryanodine receptors play an essential role in excitation-contraction coupling in striated muscle cells. The importance of their role is highlighted by the large number of regulators they have and by the consequences that individual point mutations can have on their resulting activity and the health of those who suffer from them. In addition to the most recent structural research, we have also reviewed here some of the diseases arising most frequently from the disruption of the skeletal and cardiac receptors together with the structural and functional consequences of some of the mutations giving rise to them.

Although much has been done, even more still remains unknown about the detailed operation and regulation of these molecules. Ryanodine receptors represent a challenge due to their size, complexity and physiological importance: they are connected with many cellular processes and, as a consequence, interact with many cellular proteins and other ligands, often in a specific cascade or in tandem. High-resolution structures of several RyR domains are still lacking. RyR2, which was shown to be more flexible than RyR1 by EM, in particular requires extra study, both by itself and in complex with some of its regulatory ligands. This would allow more details to be understood regarding both its structure and its physiological role. The differences between the mammalian RyR isoforms need to be better clarified as well as how they are activated in particular tissues. In this respect, RyR3 especially requires more attention. It would also be interesting to elucidate the differences between the RyR isoforms in mammals and their homologs in other animals. Research into how mutant forms of RyRs that are associated with muscular dystrophies presents an ongoing challenge: in most cases it is still unclear how the given mutations influence or initiate a particular disease. To answer such complex questions will require a multidisciplinary approach involving in vitro, in vivo and in silico studies. Structural research and structure-based drug design will certainly be an important part of it. All this knowledge should provide the foundation for establishing new medical treatments, likely individualized, for curing or at least treating the diseases with minimal side-effects.

## Figures and Tables

**Table 1:** High resolution wild-type structures of the individual domains of RyR1, RyR2, and RyR3 determined by X-ray crystallography or NMR.

PDB ID	Residues	Fragment Length	Isoform	Domain	Source	Ref.
3hsm	1–210	213	RyR1	NTD:A	*Oryctolagus cuniculus*	[158]
2xoa	1–559	559	RyR1	NTD	*Oryctolagus cuniculus*	[155]
3ila	9–205	197	RyR1	NTD:A	*Oryctolagus cuniculus*	[159]
4i96	217–536	320	RyR1	NTD:BC	*Oryctolagus cuniculus*	[156]
5c30	857–1054	201	RyR1	Repeat1–2	*Oryctolagus cuniculus*	[162]
4p9j	1070–1246	180	RyR1	SPRY2	*Oryctolagus cuniculus*	[163]
3rqr	2733–2940	227	RyR1	Repeat3–4	*Oryctolagus cuniculus*	[167]
4ert	2734–2940	210	RyR1	Repeat3–4	*Oryctolagus cuniculus*	[164]
2bcx	3614–3643	30	RyR1	central	*Oryctolagus cuniculus*	[165]
2k2f	3616–3627	12	RyR2	central	*Rattus norvegicus*	[166]
3im5	1–217	217	RyR2	NTD:A	*Mus musculus*	[159]
4l4h	1–547	547	RyR2	NTD	*Mus musculus*	[160]
4jkq	1–606	606	RyR2	NTD	*Homo sapiens*	[157]
2mc2	10–224	219	RyR2	NTD:B,C	*Mus musculus*	[161]
5c33	650–844	198	RyR2	SPRY1	*Mus musculus*	[162]
4p9i	1080–1253	174	RyR2	SPRY2	*Mus musculus*	[163]
4etv	2699–2904	209	RyR2	Repeat3–4	*Mus musculus*	[164]
6mm6	2699–2904	209	RyR2	Repeat3–4	*Mus musculus*	[168]
4erv	2597–2800	207	RyR3	Repeat3–4	*Homo sapiens*	[164]

Note: 2mc2 and 2k2f were determined by NMR. All other structures were determined by X-ray crystallography.

## Abbreviations

The following abbreviations are used in this manuscript:

RyR	ryanodine receptor
EC	Excitation-Contraction
SR	Sarcoplasmic Reticulum
LCC	L-type voltage-gated Calcium Channels
DHPR	1,4-dihydroxypyridine receptors
CaM	Calmodulin
CaMKII	Calmodulin Kinase II
PKA	Protein Kinase A
MH	Malignant Hyperthermia
CCD	Central Core Disease
MMD	Multi-Minicore Disease
CPVT1	Catecholaminergic Polymorphic Ventricular Tachycardia, type 1
ARVC/D2	Arrhythmogenic Right Ventricular Cardiomyopathy/Dysplasia, type 2
PVT	Polymorphic Ventricular Tachycardia
SIDS	Sudden Infant Death Syndrome
SCD	Sudden Cardiac Death
LVNC	Left Ventricular Non-Compaction
ER	Endoplasmic Reticulum
NTD	N-Terminal Domain
CTD	C-Terminal Domain
TM	TransMembrane region
VSC	Voltage-Sensor-like domain, Cytoplasmic
HD1,2	Helical Domain, parts 1 and 2
EM	Electron Microscopy
